# Gender disparity in tuberculosis cases in eastern and western provinces of Pakistan

**DOI:** 10.1186/1471-2334-12-244

**Published:** 2012-10-05

**Authors:** Omara F Dogar, Sarwat K Shah, Abrar A Chughtai, Ejaz Qadeer

**Affiliations:** 1 Research and Development Unit, Social and Health Inequalities Network, House 862, Street 13-C, E-11/4, NPF, Islamabad, Pakistan; 2 Research and Development Department, Health Services Academy, National Institute of Health, Chak Shehzad, Islamabad, Pakistan; 3 National Tuberculosis Control Program, Zakia Aziz Plaza, Blue Area, Islamabad, Pakistan

**Keywords:** Case notification rate, Male-to-female ratio, New sputum-smear positive, Sex-specific

## Abstract

**Background:**

Although globally, the number of notified TB cases is higher for males, a few countries in the Eastern Mediterranean Region (Afghanistan; Lebanon; Iran and Pakistan) of the World Health Organization have a relatively higher number of female cases. Pakistan ranks fifth amongst the highest TB burden countries and poses a rich ground for exploratory research to address the gender differences in TB cases. It is uniquely neighboured by India on the East, having higher number of cases in males than in females, and by Afghanistan and Iran on the West, having higher number of cases in females than in males. The objective is to see whether these gender differences are evenly distributed across the country or vary by geographies, to enable effective targeting of TB control strategies.

**Methods:**

Cross-sectional analysis was carried out on secondary data, obtained from National Tuberculosis Program. Disaggregated at the provincial level, the sex-specific case notification rates (CNR) were calculated and trends over a 10-year span (2001–2010) were examined. Sex-specific differences for the four Pakistani provinces were analyzed using chi-square test and odds ratios with corresponding confidence intervals. Cumulative countrywide sex-specific notification rates were used as the reference group.

**Results:**

The trends for 2001–2010 in the western provinces of Pakistan show higher female CNR as compared to those seen in the eastern provinces having slightly higher male CNR. The proportions of female notified TB cases are approximately twice as high in the western provinces when compared to the eastern provinces and Pakistan over all.

**Conclusions:**

These findings suggest that females are particularly affected by TB disease burden in the west parts of Pakistan. This gender disparity requires a coordinated regional and international effort to further explore triggers and moderators of increased acquisition and progression of TB disease among females in the region to guarantee effective TB control.

## Background

Each year an estimated 8·8 million new cases of tuberculosis (TB) occur worldwide, 81% of which are contributed by the 22 high TB burden countries alone. Globally, the notification rates of TB cases vary across most countries, with males being predominantly greater than females [1]. The male to female ratio (MFR) of notified new sputum smear positive (NSS +ve) TB cases for various World Health Organization’s (WHO) regions range from 1·35: 1·00 in Africa, 1·49: 1·00 in Americas, and 2·03: 1·00 in South-East Asia to 2·16: 1·00 in Europe [2]. However, the Eastern Mediterranean Region (EMR) does not conform to this global MFR; thus having a few countries with higher rates of notified NSS+ TB cases in females, including Afghanistan (MFR= 0·50: 1·00), Lebanon (MFR= 0·70: 1·00) and Iran (MFR= 0·90: 1·00) [1].

Pakistan falls among the five countries having highest TB burden in the world, with an estimated incidence of 231 new TB cases per 100 000 population, each year. TB is responsible for 5.1% of total national disease burden in Pakistan with approximately 47% of the pulmonary TB cases and 38% of all TB cases comprising of smear positive pulmonary TB [3]. It contributes about 63% of the TB burden in the EMR with a ^a^Case Notification Rate (CNR) [4] of 60 per 100 000 population for NSS +ve TB cases [1,5,6]. Overall, Pakistan shares similarities with the global MFR for the notified NSS +ve TB cases. However, when disaggregated by age and sex it shows a higher proportion of CNR for females in the reproductive age group (including 45 years of age) relative to males, with a sharp cross over, to higher CNR in males above 45 years of age [5]. Sex ratio (M/F) of Pakistan in general population, is 1.07: 1.00, according to the projection for 2010, ranging from 1·05:1·00- 1·09:1·00 for ages up to44 years and 0·93:1·00 for ages 65–69 years [7]. However, highest number of notified TB cases are seen in the age groups 15–34 in both males and females, disproportionately more in females (20-30%) [8]. A similar sex disparity in TB CNR is seen in Iran, adjacent to Pakistan and Afghanistan, with MFR of 1·00: 1·50 for the notified TB cases [2].

The sex disparity observed in notified TB cases in these EMR countries raises concern for the global audience as well as the regional and local authorities, making it eminent to understand the factors contributing to higher rates of TB spread or activation in females. Pakistan poses a rich territory for future research into the topic as it is neighboured by Afghanistan and Iran on the west, both of which have higher female notified TB cases than males (MFR: 0·50: 1·00 & 0·90: 1·00, respectively) compared to India on the east, where notified TB cases are higher in males than females (MFR: 2·30: 1·00) [1,8]. Although the reasons for these disparities span a wide array of hypotheses, this study begins by looking at the geographic distribution of CNR of NSS+ve TB across the administrative units of Pakistan. The aim is to see whether these gender differences are evenly distributed across the country or vary by geographies, to enable effective targeting of TB control strategies.

## Methods

Cross-sectional analysis was carried out on national data of notified NSS +ve TB cases for 2001 to 2010. This data is not publicly available and was obtained with permission from the National Tuberculosis Control Program (NTP), Pakistan. The case notification data is routinely collected both from public (primary, secondary and tertiary health care) and private health centres run by the NTP, embedded in the health system infrastructure of the country at provincial level, further divided into the districts and union councils comprising the cities and villages. The private health centres were included in 2007 after the Public Private Mix (PPM) launch. The PPM was started as a pilot project and is being increasingly implemented in the majority of districts in all four provinces. However, Khyber Pakhtunkhwa (KPK) and Baluchistan have the least PPM coverage while administrative regions and tribal areas still have no coverage [9].

The case notification data is collected by NTP on a quarterly basis, through standardized recording and reporting tools. Sex-specific data on NSS +ve pulmonary TB cases recorded in the TB07 form (the quarterly reporting form) for the past ten years (2001–2010) were used in the analysis. Data for KPK for the year 2001 were not available, so the trends and statistical analysis for this province were restricted to years 2002 to 2010.

Population estimates were accessed from the database of Ministry of Population Welfare (Government of Pakistan), to calculate sex-specific notification rates of NSS +ve TB cases. The male-to-female sex-ratio of the general population for each province (1·05: 1·00- Punjab, 1·11:1·00- Sindh, 1·14:1·00 Baluchistan, and 1·04:1·00- KPK)) and Pakistan (1·07:1·00) was applied to the respective directly observed treatment short-course (DOTS) population for the given year to determine sex-specific CNR over a 10-year span (2001–2010) and trends were examined [10]. DOTS coverage is defined as the population living in administrative areas where DOTS services are available. This indicator serves as a proxy for people with access to DOTS [3].

Sex-specific differences in proportion of notified cases for the four provinces were further analyzed using Chi-square test and odds ratios with corresponding confidence intervals. The MFR of notified cases in each province for the respective year, and the year-wise comparison of the proportions of male and female notified cases were determined. Pakistan’s aggregate data on notified TB cases was taken as the reference for statistical comparisons with the provincial male and female proportions of TB cases. All analyses were carried out using SAS version 9·2 (Cary, NC; USA).

This is a non-funded study. De-identified data for the study was provided by the NTP, Pakistan. None of the authors have been paid by any company. Ethics committee approval was not required as data utilized for the purpose of this study were collected as part of routine TB surveillance by the NTP.

## Results

### Trends in TB case notification rates (2001–2010) of the four provinces and Pakistan overall

**Figure 1:**
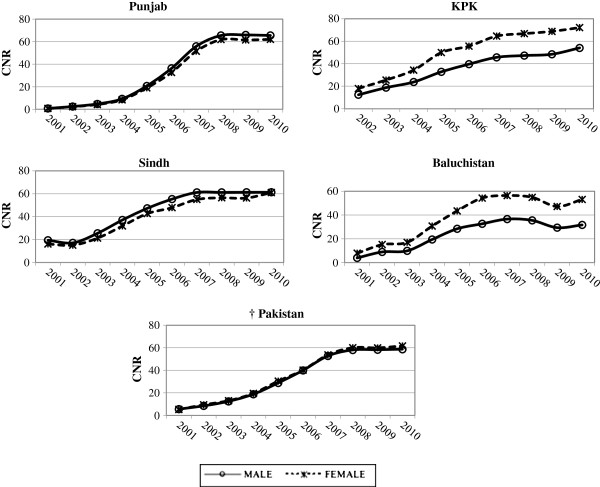
**Trends of CNR by Sex for Provinces and Pakistan (2001–2010).** * Data is from 2002 – 2010 †Data includes the North-western Regions in addition to the Provinces CNR: Case notification rate.

The study population consisted of 571958 NSS +ve cases, from 2001 to 2010, reported from the whole of Pakistan to the National TB Program. Out of these, 292551 were males and 279407 were females. During this time, CNR for NSS +ve were nearly equal for males and females in Pakistan. However, when disaggregated at provincial level, the proportion of female notified cases in the two western provinces were higher than male notified cases, discordant with the findings for the eastern provinces, where male notified cases were higher than female notified cases (Figure 1).

In the eastern provinces the gender pattern in notification rates persisted over time with a slight predominance of male notified cases resembling the trends for the whole of Pakistan. Contrary to this higher number of female notified cases relative to males were observed in the western provinces. The western provinces showed a uniformly widening gap in the male and female CNRs, from 2001, which levelled after 2005 with a difference of approximately 20% between them. Overall, the CNRs of male and female cases increased over time (2001–2010).

### Sex- specific TB case notifications- comparison of the four provinces with Pakistan overall

**Figure 2:**
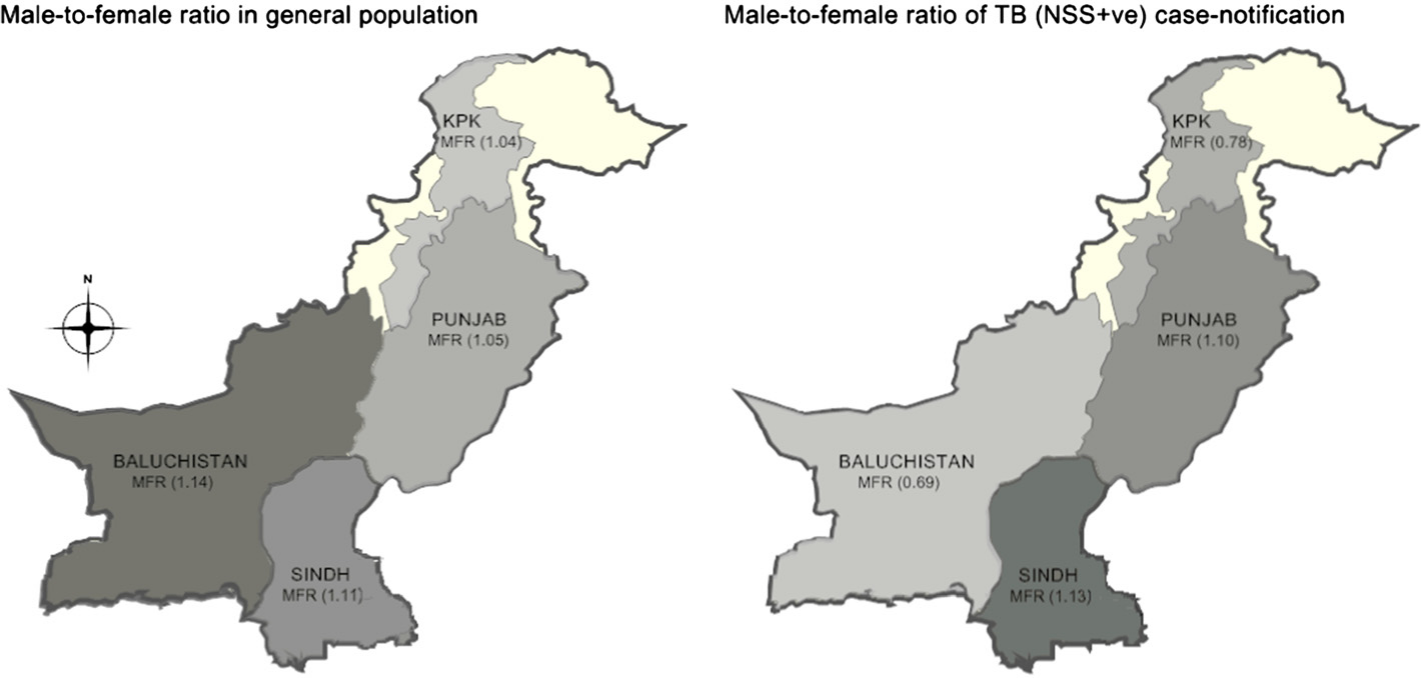
**Male-to-female sex ratio in the general population and new smear positive TB cases notified by provinces (2010).** KPK: Khyber-pakhtoonkhwa province; MFR: male-to-female ratio; TB (NSS+ve): new sputum smear positive tuberculosis * Note: Darker shades represent higher MFR.

Over a ten year period, the MFR of notified cases for the Eastern provinces remained, on average, 1·27:1·00 (range: 1·13 – 1·35) and 1·12:1·00 (range: 1·01- 1·16) for Sindh and Punjab, respectively. However, for the western provinces, the average MFR was 0·74:1·00 (range: 0·68 – 0·78) and 0·70:1·00 (range: 0·57 – 0·75), for KPK and Baluchistan, respectively. The geographic distribution of these differences is evident (Table 1). Figure 2, shows the MFR for the general population and for the notified TB cases by provinces for 2010 where darker shades represent higher MFR.

**Table 1:** TB (NSS+ve) Case Notification by sex, from 2001–2010

**Years**		*Pakistan	**Punjab**	‡**OR (**† **95**% **CI)**	**Balochistan**	**OR (95**% **CI)**	**Sindh**	**OR (95**% **CI)**	**KPK**	**OR (95**% **CI)**
**2001**	Males	3258	306		150		2280		-	-
	Females	2865	302	1·12 (0·95-1·33)	262	1·99 (1·62- 2·44)	1684	0·84 (0·77-0·91)		
	¤ MFR	(1·14)	(1·01)		(0·57)		(1·35)			
**2002**	Males	6521	1011		347		3029		1239	
	Females	6725	917	0·87 (0·80-0·97)	512	1·43 (1·24 -1·65)	2372	0·76 (0·71-0·81)	1720	1·35 (1·24-1·46)
	MFR	(0·97)	(1·10)		(0·68)		(1·28)		(0·72)	
**2003**	Males	9820	1985		391		4634		1907	
	Females	9504	1720	0·90 (0·83-0·96)	585	1·55 (1·36 -1·76)	3431	0·76 (0·73-0·81)	2492	1·35 (1·26-1·44)
	MFR	(1·03)	(1·15)		(0·67)		(1·35)		(0·77)	
**2004**	Males	15113	3962		783		6851		2465	
	Females	14520	3459	0·91 (0·86-0·96)	1076	1·43 (1·30 -1·57)	5224	0·79 (0·76-0·83)	3422	1·45 (1·37-1·53)
	MFR	(1·04)	(1·15)		(0·73)		(1·31)		(0·72)	
**2005**	Males	23661	9039		1168		8915		3473	
	Females	23086	7986	0·91 (0·87-0·94)	1552	1·36 (1·26 -1·47)	7140	0·82 (0·79-0·85)	5081	1·50 (1·43-1·57)
	MFR	(1·02)	(1·13)		(0·75)		(1·25)		(0·68)	
**2006**	Males	33461	16188		1367		10646		4278	
	Females	31126	13998	0·93 (0·90-0·96)	1976	1·55 (1·45 - 1·67)	8166	0·82 (0·80-0·85)	5776	1·45 (1·39-1·51)
	MFR	(1·08)	(1·16)		(0·69)		(1·3)		(0·74)	
**2007**	Males	45123	25405		1564		12006		5029	
	Females	42442	22523	0·94 (0·92-0·96)	2094	1·42 (1·33 - 1·52)	9582	0·85 (0·82-0·87)	6838	1·45 (1·39-1·50)
	MFR	(1·06)	(1·13)		(0·75)		(1·25)		(0·74)	
**2008**	Males	50587	30374		1519		12253		5308	
	Females	48341	27558	0·95 (0·93-0·97)	2040	1·41 (1·31 - 1·50)	10030	0·86 (0·83-0·88)	7209	1·42 (1·37-1·48)
	MFR	(1·05)	(1·10)		(0·74)		(1·22)		(0·74)	
	Males	51974	31199		1368		12517		5547	
	Females	49354	27963	0·94 (0·92-0·96)	1908	1·47 (1·37-1·58)	10193	0·86 (0·83-0·88)	7572	1·44 (1·39-1·49)
	(MFR)	(1·05)	(1·12)		(0·72)		(1·23)		(0·73)	
**2010**	Males	53033	31478		1474		12775		6191	
	Females	51444	28644	0·94 (0·92-0·96)	2147	1·50 (1·40-1·61)	11274	0·91 (0·88-0·94)	7945	1·32 (1·28-1·37)
	MFR	(1·03)	(1·10)		(0·69)		(1·13)		(0·78)	

† CI= Confidence Interval.

‡ OR= Odds Ratio.

* Pakistan has been taken as the reference, for comparison.

¤ Male-to-female ratio of notified TB cases.

The proportion of female notified TB cases exceeds male cases, from 36% up to 99% (range of OR: 1·36- 1·99), in Baluchistan (Table 1). Similarly in KPK the proportion of female notified TB cases are from 30% up to 50% (range of OR: 1·32- 1·50) more than males when compared to the reference group, over a ten year period. The differences in proportions for both the western provinces are statistically significant. In the two eastern provinces, male and female CNRs are proportionate, as evident from the odds ratios.

## Discussion

The results show higher TB case notifications among females in the western provinces of Pakistan in comparison to the eastern provinces. These findings are in accord with the CNR of the bordering countries, Afghanistan and Iran on the west and India on the east of Pakistan. Holistically, the CNRs of male and female cases have shown an increasing trend over time (2001–2010) consistent with the significant increase in the coverage of DOTS population [3].

Tuberculosis kills more women each year than any other infection, being the third cause of mortality and morbidity combined, in women of reproductive age group in the developing world [11]. Women’s risk of TB disease may be explained by the differential exposure to tubercle bacilli which might be attributable to sexual division of labour, cultural seclusion practices and socialization patterns [12]. The differences between type and concentration of sex-steroid and non-sex steroid hormones secretion have also been shown to play a role and lead to higher immune response to TB in males [13].

The development of active disease and the progression of latent to active tuberculosis are noted to be higher in females [13-15]. This is further supported by an 8 year cohort study where 130% higher risk of progression was seen in women of the reproductive age group (10–44 years) [15]. People who acquire new infection progress to disease quicker than those who have older infections; as noticed by Holmes et. al, women had lower ARTI (annual risk of tuberculosis infection) before the reproductive age group than men, creating a fresh pool of susceptible population, to be newly infected with TB, who progress to disease rapidly [14]. The same phenomenon is observed in Afghanistan, where ARTI was lower in adolescents and young women but disease progression was almost 3 times that of males [16].

Increased rates of progression might be seen in societies where women have worse health than men in terms of nutrition [12]. Evidence exists for higher prevalence of TB disease (2.8 times higher) among strict vegetarians relative to those who eat a varied diet [12,17,18]. Several recent studies have linked vitamin D deficiency with increased risk of tuberculosis [17,18]; a meta-analysis showed pooled effect size of 0.68 for vitamin D levels in persons with TB compared to healthy controls [17]. Vitamin D influences immune response to TB by promoting the formation of phagolysosomes and formation of anti-microbial peptide LL-37, that has direct bactericidal activity and an immune regulating function [17]. Another interesting point highlighted in these studies is the rapid killing of tubercle bacilli by sunlight [12] and the lack of exposure to sunlight as a contributor to vitamin D deficiency [18]. The western provinces of Pakistan, especially Baluchistan is dry and sandy and the men spend more time in sunlight daily (working in fields and travelling on foot); in comparison women spend more time completing chores inside homes [19]. Women are the sole caretakers of the sick at home but are not taken care of by their husbands if they fall sick, making them more vulnerable to contract TB [11]. They also tend to spend more time indoors than outside their houses, being exposed to a higher infectious dose of mycobacterium TB from the sick at home [11,19].

Tobacco smoking (both active and passive) and other smoke exposures like biomass fuels used for cooking fire and air pollution from coal fires have been associated with TB [19]. The western provinces are comprised of a predominantly rural population (83 and 76 percent, respectively) in comparison to the eastern provinces (69 and 51 percent, respectively) [20]. Women are disproportionately exposed to cooking fire, passive smoke and habitual tobacco smoke in the form of ‘hookah’ in rural parts of Pakistan that may partly explain female excess in TB cases in western provinces but this need to be established with evidence from future research [21].

Considering an evolving HIV epidemic in concentrated high risk groups in Pakistan, it is highly likely that the prevalence of HIV in TB patients will also have increased with the maturation of this epidemic; a study done in rural parts of Pakistan in Sindh province found HIV prevalence of 0.5% in TB patients which is more than the estimated prevalence of < 0.1% in the general population [22].

Physiological and immune status alterations in pregnancy allow mothers to tolerate genetically different foetal tissues during pregnancy; this is mediated through impaired cell-mediated immunity that in turn increases susceptibility to infections like tuberculosis during pregnancy [23]. It might be a possibility that early marriages and higher fertility rates partly contribute to the observed gender disparity in western provinces of Pakistan.

Identical trends for sex differences in TB notification rates were observed in Norway, Denmark, England and Wales in mid-20th century leading one to think reasons other than socio-cultural, lifestyle and ethnicity factors that might influence the TB disease dynamics [14]. A commonality amongst these countries and Pakistan is that all of the regions are or were subject to disasters (natural and man-made) leading to mass displacement of populations. The western provinces of Pakistan accommodate a large pool of externally displaced people due to the refugee influx from Afghanistan. In addition, Pakistan’s western provinces have faced movements of masses of internally displaced people due to conflicts, devastating earthquakes and floods in the recent decade.

We presented CNR as a measure of disease burden in this study because true incidence of TB is currently not available for Pakistan. Although CNR under-represents the disease burden for any country [4], it is often a good approximation of incidence in settings where health care system, diagnostics and reporting of tuberculosis is of reasonable quality [9]. If CNR for all types of TB was used instead as an indicator for disease burden, the numerator (number of total TB cases) might have been influenced by the capacity to diagnose extra-pulmonary and sputum smear negative pulmonary cases, for example due to the availability of diagnostic methods or the skill of the physician to interpret chest X-ray abnormalities etc.) [4]. Therefore, only NSS+ve TB cases were used to derive CNR as it uses a single objective method (sputum microscopy) [4]. An indicator used for the quality assurance of sputum smear microscopy is the percentage of supervisory visits of laboratories at district and sub-district levels by the reference laboratory officers out of the planned for each province [3]. The percentage of supervisory visits was more than 90% for Punjab and Sindh, while it was 86% for KPK and 62% for Balochistan, in 2010 [3].

In general, socio-economic and cultural factors seem to play an essential role in determining overall gender differences in rates of infection and progression to disease. Pakistan shares socio-cultural and ethnic similarities with other countries in the region that require further evidence base to infer linkages with the observed sex disparity.

## Conclusions

These findings strongly suggest that females are particularly affected by TB disease burden in the west parts of Pakistan. Either these differences are due to the exposure of females to certain factors predisposing them to the increased acquisition and/or progression of latent to active disease in comparison to males, or it is the confounding effect of increased notification of female cases. This requires further explanation through evidence-based research which can consequently be translated into policy leading to the effective implementation of TB control measures in practice to address this gender disparity.

## Endnotes

^a^CNR: The annual reported number of TB cases divided by the total population in the specified area per 100 000.

## Abbreviations

TB: Tuberculosis; NSS +ve: New sputum smear positive; MFR: Male-to-female ratio; WHO: World Health Organization; EMR: Eastern-Mediterranean region; CNR: Case notification rate; PPM: Public private mix; DOTS: Directly observed treatment short-course; KPK: Khyber-pakhtoonkhwa; NTP: National Tuberculosis Program; ARTI: (annual risk of tuberculosis infection).

## Competing interests

The authors declare that they have no competing interests.

## Authors’ contributions

OFD- 1) Conceived the idea and contributed to conception, analysis and interpretation of data, 2) Drafted the main sections of the manuscript, and, 3) Approved the version to be published. SKS- 1) Substantially participated in conception and design, acquisition and interpretation of data 2) drafting the results and methods (in parts) section of the manuscript and revising other parts of the paper critically for important intellectual content, and, 3) Final approval of the version to be published. AAC- 1) Substantially contributed to data acquisition and interpretation, 2) Revising it critically for technical programmatic content, and, 3) Final approval of the version to be published. EQ- 1) Provided the data, 2) participated in revising the manuscript critically for important intellectual content, and, 3) final approval of the version to be published. All authors read and approved the final manuscript.

## Pre-publication history

The pre-publication history for this paper can be accessed here:

http://www.biomedcentral.com/1471-2334/12/244/prepub
